# Bactericidal and Anti-biofilm Effects of Polyhexamethylene Biguanide in Models of Intracellular and Biofilm of *Staphylococcus aureus* Isolated from Bovine Mastitis

**DOI:** 10.3389/fmicb.2017.01518

**Published:** 2017-08-11

**Authors:** Nor F. Kamaruzzaman, Stacy Q. Y. Chong, Kamina M. Edmondson-Brown, Winnie Ntow-Boahene, Marjorie Bardiau, Liam Good

**Affiliations:** ^1^Department of Pathology and Pathogen Biology, Royal Veterinary College London, United Kingdom; ^2^Centre for Expertise in the Treatment and Management of Water (CEBEDEAU) Liège, Belgium

**Keywords:** mastitis, *S. aureus*, intracellular bacteria, antimicrobial polymer, enrofloxacin, polyhexamethylene biguanide

## Abstract

*Staphylococcus aureus* infection is a common cause of mastitis, reducing milk yield, affecting animal welfare and causing huge economic losses within the dairy industry. In addition to the problem of acquired drug resistance, bacterial invasion into udder cells and the formation of surface biofilms are believed to reduce antibiotic efficacy, leading to treatment failure. Here, we investigated the antimicrobial activities of enrofloxacin, an antibiotic that is commonly used in mastitis therapy and polyhexamethylene biguanide (PHMB), an antimicrobial polymer. The antimicrobial activities were tested against intracellular *S. aureus* in infected Mac-T cells (host cells). Also, fluorescein-tagged PHMB was used to study PHMB uptake and localization with *S. aureus* within the infected Mac-T cells. Anti-biofilm activities were tested by treating *S. aureus* biofilms and measuring effects on biofilm mass *in vitro*. Enrofloxacin and PHMB at 15 mg/L killed between 42 to 92 and 99.9% of intracellular *S. aureus*, respectively. PHMB-FITC entered and colocalized with the intracellular *S. aureus*, suggesting direct interaction of the drug with the bacteria inside the host cells. Enrofloxacin and PHMB at 15 mg/L reduced between 10 to 27% and 28 to 37% of biofilms’ mass, respectively. The half-maximal inhibitory concentrations (IC_50_) obtained from a cytotoxicity assay were 345 ± 91 and 21 ± 2 mg/L for enrofloxacin and PHMB, respectively; therefore, both compounds were tolerated by the host cells at high concentrations. These findings suggest that both antimicrobials are effective against intracellular *S. aureus* and can disrupt biofilm structures, with PHMB being more potent against intracellular *S. aureus*, highlighting the potential application of PHMB in mastitis therapy.

## Introduction

Bovine mastitis causes huge economic losses in the dairy industry (Huijps et al., 2008). The disease has been identified as the most common cause of morbidity in adult dairy cows in the United States (USDA, 2007). Mastitis is manifested by inflammation of the mammary gland, triggered by bacteria invasion through the teat canal (Gruet et al., 2001). A range of Gram-negative and -positive bacteria, mycoplasmas and algae are associated with mastitis. The majority of cases appear to be caused by *Eschericia coli (E. coli), Streptococcus uberis (S. uberis)*, and *S. aureus* (Cheng et al., 2010; Zadoks et al., 2011). *S. aureus* is associated with up to 30% of bovine mastitis cases, causing sub-clinical and chronic infections in the mammary gland (Weber et al., 2006; Halasa et al., 2007). *S. aureus* produces toxins that can induce necrosis (cell death) of milk secretory tissues, reducing milk production and food safety (Zhao and Lacasse, 2008).

The recommended treatment for mastitis is administration of antibiotics, with beta lactams (e.g., penicillin), tetracyclines and enrofloxacin among the most widely used drugs. However, cure rates for mastitis caused by *S. aureus* range from 4 to 92% (Barkema et al., 2006). Acquired resistance toward these antibiotics has reduced therapy success rates (Pyörälä and Pyörälä, 1998). Also, *S. aureus* is known to invade and localize inside host alveolar cells and macrophages (Hébert et al., 2000; Brouillette et al., 2003). Poor accumulation of beta lactams inside the host cells leads to in-complete clearance of the bacteria (Tulkens, 1991). Moreover, recent evidence by Silva et al. (2014) demonstrated that enrofloxacin induces *E. coli* internalization by bovine mammary alveolar cells.

The treatment for *S. aureus* mastitis is further complicated by the formation of bacterial biofilms on the mammary gland. Biofilms are structured communities of bacterial cells enclosed in a self-produced polymeric matrix and adherent to an inert or living surface (Melchior et al., 2006). The extracellular polymeric substances (EPS) are built of polysaccharide, protein, extracellular DNA, and lipids (Arciola et al., 2015; Roy et al., 2017). Bacteria in biofilms are up to 1000-fold less susceptible to antimicrobials (Ceri et al., 1999). Reduced susceptibility is believed to be due to restricted diffusion of drugs across the biofilm structure (Melchior et al., 2006) and physiological changes in the bacteria resident within biofilms (e.g., slower growth) may further reduce susceptibility (Costerton et al., 1999). Furthermore, a recent study demonstrated that enrofloxacin at sub-minimum inhibitory concentrations induces biofilm formation by *E. coli* and *S. aureus* (Costa et al., 2012; Seixas et al., 2015). Together; antimicrobial resistance, host cellular invasion and formation of biofilms, can lead to therapeutic failures, subsequently, causing persistent mastitis in the animal. Therefore, there is an urgent need to find alternative therapies to reduce the burden of the disease.

**FIGURE 1:**
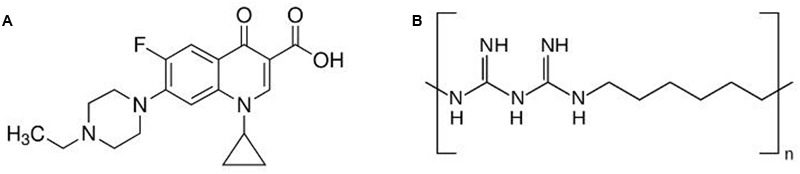
Structure of **(A)** enrofloxacin and **(B)** polyhexamethylene biguanide (PHMB). Enrofloxacin is a fluoroquinolone with mw of 359.4 g/mol. PHMB is a cationic polymer of repeating hexamethylene biguanide groups, with *n* average = 10–12 (*n* is the number of structural unit repeats) and molecular weight (mw) 3025 g/mol.

No new antimicrobials effective against mastitis have been developed during recent decades, and there is increasing concern about antimicrobial resistance in food systems. Given that mastitis therapy is typically administered locally, there may be scope for using antimicrobial polymers in therapy. Antimicrobial polymers appear much less susceptible to acquired resistance, and they can be surprisingly effective against intracellular pathogens. For example, we recently reported that a cationic polymer, polyhexamethylene biguanide (PHMB) (**Figure 1**), effectively killed intracellular *S. aureus* inside keratinocytes and the parasite *Leishmania* in macrophages, through a dynamin dependent uptake mechanism (Firdessa et al., 2015; Kamaruzzaman et al., 2016). Therefore, we considered that PHMB might also be effective against intracellular *S. aureus* within bovine mammary epithelial cells. Similarly, we considered that the cell penetration and DNA binding properties of PHMB (Chindera et al., 2016) may enable this polymer to enter or destabilize biofilm structures.

Here we tested the potential for PHMB to enter bovine mammary epithelial cells and kill *S. aureus* that are sequestered within the host cells. Three recent clinical isolates of *S. aureus* from clinical cases of bovine mastitis were included. Also, we tested anti-biofilm effects against the clinical isolates. We use the term anti-biofilm to refer to “a natural or induced processes, leading to reduction of bacterial biomass through the alteration of biofilm formation, integrity and in quality” as suggested by Miquel et al. (2016). For a comparison, we included enrofloxacin (**Figure 1**), a fluoroquinolone that can enter mammalian cells and has anti-biofilm activity (Van Bambeke et al., 2005; Benoit et al., 2010).

## Materials and Methods

### Bacterial Strains and Growth Conditions

*Staphylococcus aureus* strain 15 AL was obtained from Dr. Shan Goh, Royal Veterinary College, London strain 59 and 204 was obtained from Dr. Marjorie Bardiau, University of Brighton. The three strains were selected because they produce biofilms and are recent field isolates (Bardiau et al., 2013; Prenafeta et al., 2014). Bacteria were grown in Mueller Hinton Broth (MHB, Sigma–Aldrich, United Kingdom) followed by incubation at 250 rpm (for liquid cultures), at 37°C for 18 h.

### Eukaryotic Cell Lines and Growth Conditions

Mac-T cells used as the host for intracellular infection were obtained from Dr. Amanda Gibson (Royal Veterinary College). The cells were maintained in DMEM with 10% FBS (Sigma–Aldrich, United Kingdom), supplemented with 5% penicillin-streptomycin (Sigma–Aldrich, United Kingdom) and 1% insulin. Cells were maintained at 37°C in 5% carbon dioxide.

### Determination of Minimum Inhibitory Concentrations (MIC)

Enrofloxacin were purchased from Sigma–Aldrich, United Kingdom. PHMB and fluorescein-tagged PHMB (PHMB-FITC) were from Tecrea Ltd, United Kingdom. All antimicrobials were prepared in stock solution at 10, 000 mg/L. Enrofloxacin was dissolved in 0.02 M sodium hydroxide and PHMB were dissolved in sterile distilled water.

minimum inhibitory concentrations (MICs) were determined using the broth microdilution method (CLSI, 2007). Briefly, a range of concentrations of antimicrobials were prepared in a 96-well microplate, followed by inoculation of bacteria to yield ∼5 × 10^5^ cfu/mL in a 250 μl final volume. The plate was then incubated at 37°C for 18 h. The lowest concentration of antimicrobial that inhibited growth of bacteria was scored as the MIC.

### Intracellular Infections of Mac-T by *S. aureus*

Intracellular infections of Mac-T cells by *S. aureus* were established using a gentamicin protection procedure as described in our previous work (Kamaruzzaman et al., 2016). Briefly, Mac-T cells were co-incubated with *S. aureus* for 3 h for cellular invasion, followed by gentamicin exposure at 200 mg/L for another 3 h to kill extracellular bacteria. Finally, cells were lysed with one ml of 0.5% Triton X-100, prepared in PBS to release the intracellular bacteria.

### Visualization of Intracellular *S. aureus* Within Bovine Mammary Epithelial Cells

Cells were grown on glass cover slips in a 12 wells plate followed by *S. aureus* infections as described above. Following gentamicin exposure to kill extracellular bacteria, cells were rinsed with PBS and fixed with 4% paraformaldehyde (Santa Cruz Biotechnology, United Kingdom). Cells were stained with 5 mg/L DAPI (Life technologies, United Kingdom) for nuclei staining and 5 mg/L Wheat Germ Agglutinin-conjugated Alexa Fluor 555 (WGA, Life technologies, United Kingdom) for membrane staining. Cover slips were mounted onto glass slides with FluorSave^TM^ (Calbiochem, United Kingdom). Images were visualized using a Leica SP5 confocal microscope using Advanced Fluorescence Software (Leica Microsystems, Milton Keynes, United Kingdom).

### Intracellular Killing Activities of *S. aureus* by Enrofloxacin and PHMB

Host cells were infected with *S. aureus* followed by incubation with gentamicin to kill extracellular bacteria. Following gentamicin exposure, cells were rinsed with PBS, and antimicrobials (enrofloxacin or PHMB in medium) were added to the wells containing the infected cells, and the plates were incubated for another 3 h. Following this procedure, the antimicrobials solutions were removed. Cells were then rinsed and lysed. Lysed cells were serially diluted and plated on nutrient agar. For each experiment, gentamicin treated cells (without enrofloxacin or PHMB treatments) were used as controls.

### Visualization and Quantification of PHMB Uptake into Mac-T Cells

Mac-T cells were grown on glass cover slips in a 12 wells plate followed by *S. aureus* infections as described above. Following gentamicin exposure to kill extracellular bacteria, cells were rinsed with PBS and fixed with 4% paraformaldehyde (Santa Cruz Biotechnology, United Kingdom). Cells were stained with 5 mg/L DAPI (Life technologies, United Kingdom) for nuclei staining and 5 mg/L Wheat Germ Agglutinin-conjugated Alexa Fluor 555 (WGA, Life technologies, United Kingdom) for membrane staining. Cover slips were mounted onto glass slides with FluorSave^TM^ (Calbiochem, United Kingdom). Images were visualized using a Leica SP5 confocal microscope using Advanced Fluorescence Software (Leica Microsystems, Milton Keynes, United Kingdom). Sequential scan Z-stacks (130 slices 1024 × 1024) were compiled at a line average of 96, using Volocity^®^ software. Three-dimensional (3D) Image Analysis Software was used for analysis and to produce 3D images. To quantify PHMB uptake into Mac-T cells, PHMB-FITC treated cells were incubated with 0.04% Trypan blue (Invitrogen, United Kingdom) in PBS for 15 minutes to quench membrane bounded PHMB-FITC, followed by flow cytometry (FACSBD machine and BDFACSDiva^TM^ software, BD Bioscience) using the FITC filter. For each sample, 10,000 gated cells were analyzed.

### Cytotoxicity Assay

Epithelial cells (4 × 10^4^ cells/well) were added to a 96-well plate and cultured with increasing concentrations of enrofloxacin or PHMB at 37°C for 24 h. Non-treated cells and medium only were used as controls. Resazurin sodium salt (Sigma–Aldrich, United Kingdom) was prepared as a stock solution at 440 μM in PBS and added to each well at 44 μM final concentration and plates were incubated for an additional 48 h. Optical density (OD) was then measured using a Tecan Infinite plate reader (Tecan group Ltd, Switzerland) at 550 and 630 nm. The OD value change (or % dye reduction) is proportional to the viable cell number and was used to calculate half maximal inhibitory concentrations (IC_50_) values based on the intercept theorem.

### Development of Biofilms

Biofilms were developed according to the protocol described by Prenafeta et al. (2014) with modifications. Briefly, *S. aureus* strains were cultured overnight in a TSB medium, supplemented with 2% glucose at 37°C in an incubator shaker. The overnight cultures were diluted to a final concentration of 10^7^ cfu/mL in a new fresh TSB medium. Aliquots of bacteria (1 mL, 10^7^ cfu/mL) were added into a 12 wells cell culture plate and further incubated in a 37°C incubator for 48 h. After 48 h, the non-adhered cultures were removed by pipette. To increase stability of the biofilms, the biofilms were heat-fixed at 60°C for 1 h. Following that, 1 mL of 0.1% crystal violet diluted in water was added to stain the biofilms for 15 min at room temperature. After that, the crystal violet solution was removed by pipette, and the biofilms were rinsed once with water. Biofilms mass was quantified by measuring optical density of crystal violet at 550 nm using spectrophotometer.

### Visualization of Biofilms Using Microscopy

*Staphylococcus aureus* was grown overnight as described above. Biofilms were developed on glass cover slips in a 12 wells cell culture plate. Following 48 h of incubation at 37°C, the non-adhered cells were removed and biofilms were heat fixed at 60°C. Biofilms were fixed with 4% paraformaldehyde followed by DAPI and WGA staining.

### Anti-Biofilms Activity of Enrofloxacin and PHMB

The biofilms were developed as described above. Following 48 h of incubation at 37°C, antimicrobials (enrofloxacin and PHMB) were slowly added to the wells, without removal of the non-adhered cells, and the plates were further incubated at 37°C for another 3 h. Following that, the medium was removed and the remaining biofilms were heat fixed, stained with crystal violet, rinsed and proceed for OD measurement. Anti-biofilm activity of antimicrobials was measured based on the percentage reduction of biofilm mass in comparison to the untreated biofilms. To determine whether bacteria within the treated were viable, the biofilms were disrupted by sonication for 5 min in a bath sonicator to release the individual cells, serial diluted in normal saline, and plated onto nutrient agar for colony counting (Bjerkan et al., 2009).

### Statistical Analysis

All experiments were performed in triplicate. Statistical analysis was performed using one-way Analysis of Variance (ANOVA) followed by Tukey tests using the statistical packages Prism 6, Version 6.0 (GraphPad Prism 6.0, San Diego, CA, United States). Data is presented as means ± standard deviation. Differences were scored as statistically significant where *p* ≤ 0.05. For histogram and graphs, error bars represent standard deviations. ^∗^*p* ≤ 0.05, ^∗∗∗^*p* ≤ 0.001, ^∗∗∗∗^*p* ≤ 0.0001, ns (not significant).

## Results

### Susceptibility of *S. aureus* Strains to Enrofloxacin and PHMB

To measure susceptibility of *S. aureus* to enrofloxacin and PHMB, MIC values were determined for all three strains tested; strain 15AL, 59 and 204. Both antimicrobials displayed potent growth inhibitory effects. The MIC values for enrofloxacin were as follows: 0.125 mg/L for strains 59 and 204, and 0.25 mg/L for strain 15A, lower than PHMB in all three strains tested. The MIC value for PHMB for all three strains tested were 1 mg/L.

### Intracellular Invasion of Mac-T Cells by *S. aureus*

**FIGURE 2:**
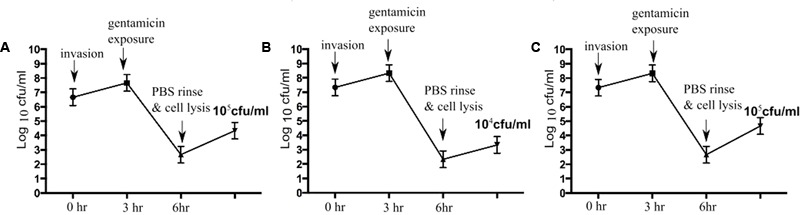
*Staphylococcus aureus* invasion in Mac-T cells. Colony forming units (cfu) of *S. aureus* following gentamicin exposure. After gentamicin exposure, lysis of Mac-T cells released approximately 10^5^ cfu/mL of *S. aureus* 15AL **(A)**, 10^4^ cfu/mL of 59 **(B)**, and 10^5^ cfu/mL of 204 **(C)**.

**FIGURE 3:**
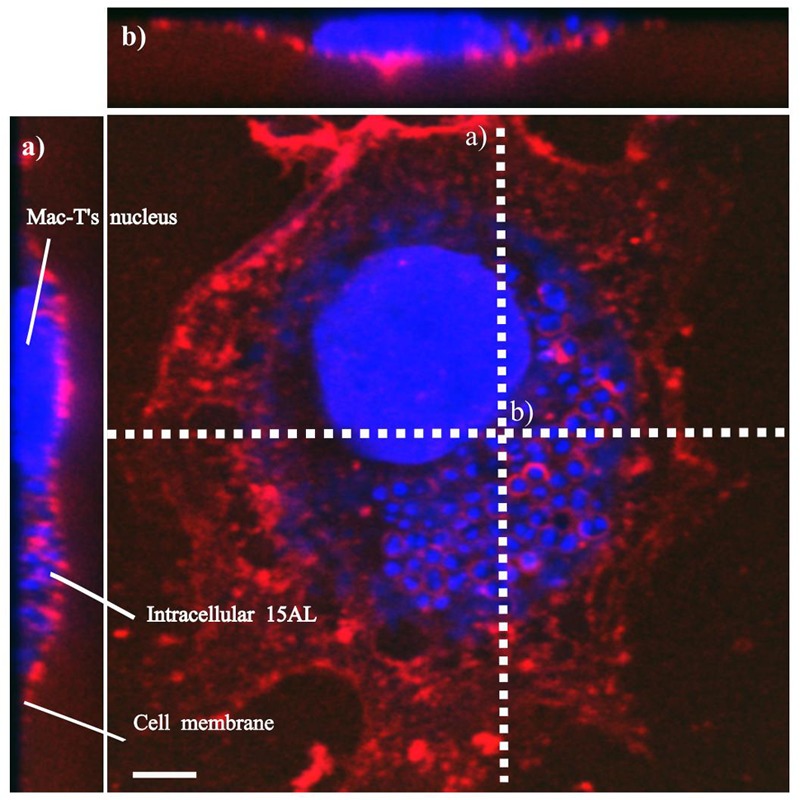
Localization of *S. aureus* in Mac-T cells. Invasion of *S. aureus* 15 AL in Mac-T cells was visualized using confocal microscopy. Prior to imaging, cells were stained with DAPI (blue) for epithelial cells and 15 AL DNA staining and WGA (red) for host cells membrane staining. Confocal microscopy *z*-stack projection moved through 139 slices across the cell. **(a)** Horizontal cross-section of Mac-T cells **(b)** Vertical cross-section of Mac-T cells. The small blue dots are *S. aureus* 15AL cells inside Mac-T cells, indicating invasion. White scale bar is 7.5 μm.

To determine bactericidal activities of enrofloxacin and PHMB against intracellular *S. aureus*, an *in vitro* intracellular infection assay was established using Mac-T cells as host. The Mac-T cell line was established from the bovine mammary alveolar primary cells (Huynh et al., 1991). It is commonly used as a model for udder epithelial cells and as a host for bacterial intracellular infections (Bayles et al., 1998; Dogan et al., 2006). Intracellular infections were performed using gentamicin protection assay as described in our previous work with keratinocytes (Kamaruzzaman et al., 2016). The same procedure used for *S. aureus* invasion in keratinocytes was reproducible in this cell culture model. All three *S. aureus* strains included in this study invaded the host cells, as indicated by their ability to evade gentamicin treatment (**Figure 2**). Gentamicin is an antibiotic that is active against extracellular bacteria, but not against intracellular bacteria, due to its polar properties and poor penetration into mammalian cells (Vaudaux and Waldvogel, 1979). *S. aureus* invasion of the host cells was further confirmed by visualization using confocal microscopy. Using three-dimensional imaging analysis, we observed localization of *S. aureus* strain 15 AL (blue dots) inside Mac-T cells membranes (red) (**Figure 3**), confirming invasion of *S. aureus* in host cells. These results show that the *S. aureus* isolated from cases of bovine mastitis are invasive.

### Bactericidal Activities Enrofloxacin and PHMB against Intracellular *S. aureus*

**FIGURE 4:**
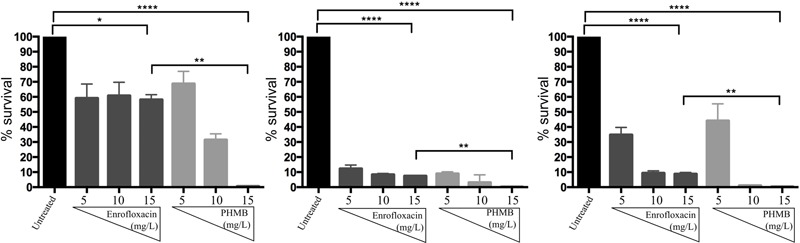
Bactericidal activities of enrofloxacin and PHMB against intracellular *S. aureus* 15 AL, 59, and 204. Mac-T cells infected with *S. aureus* strain 15 AL, 59, or 204 were either untreated or treated with increasing concentrations of enrofloxacin or PHMB. Untreated cultures were used to establish cfu values corresponding to 100% survival.

To investigate bactericidal activities of enrofloxacin and PHMB against intracellular *S. aureus*, host cells were infected with *S. aureus* and exposed to gentamicin to kill extracellular bacteria, as described above. Infected cells were treated with enrofloxacin or PHMB at 5, 10, or 15 mg/L for 3 h followed by enumeration of cfu of surviving intracellular bacteria. **Figure 4** shows the percentages of surviving intracellular *S. aureus* after treatment with enrofloxacin and PHMB, relative to the untreated infected cells. Enrofloxacin at 15 mg/L killed between 42 and 92% of intracellular *S. aureus* and PHMB at 15 mg/L killed 99.9% of intracellular *S. aureus*. Therefore, while both enrofloxacin and PHMB demonstrated activities against intracellular *S. aureus*, PHMB showed more potent and consistent bactericidal activities in comparison with enrofloxacin against intracellular *S. aureus.*

### PHMB Uptake into Mac-T Cells

**FIGURE 5:**
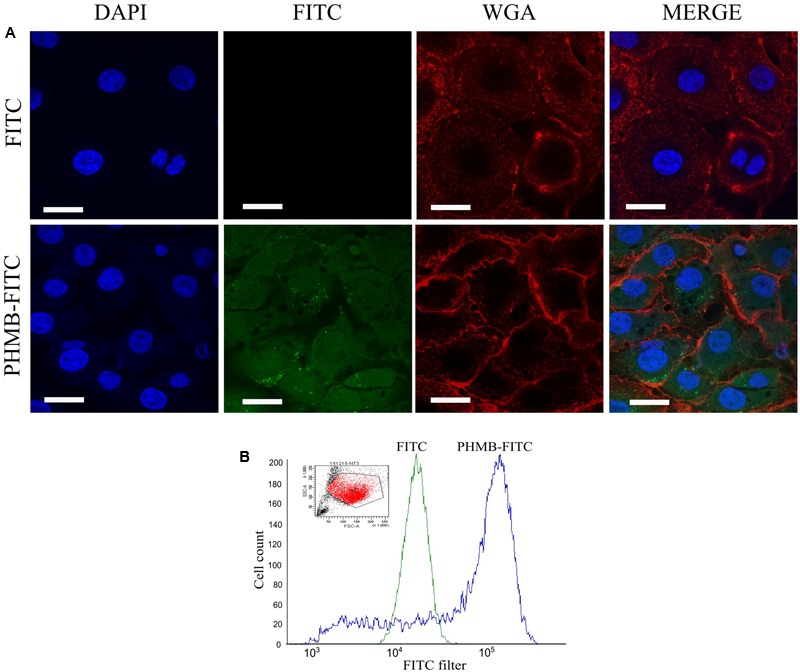
Fluorescein-tagged PHMB (PHMB-FITC) uptake into Mac-T cells. **(A)** PHMB-FITC localization visualized by confocal microscopy. Free FITC was used as control. **(B)** PHMB-FITC uptake was quantified by flow cytometry. The small image represents the population of Mac-T cells gated in the analysis. The overlay histogram image illustrates the uptake of PHMB-FITC into Mac-T cells. The green profile shows the population of cells treated with FITC and serves as a negative control. The blue profile shows the population of cells treated with PHMB-FITC. Uptake of PHMB-FITC was observed in approximately 70% of Mac-T cells.

Killing of intracellular *S. aureus* by antimicrobials normally requires drug entry into the host cells and direct contact with the bacteria. To test whether PHMB can enter Mac-T cells, PHMB-FITC was added to the cell cultures and its intracellular localization was tracked using confocal microscopy. As can be seen in **Figure 5A**, a mixture of predominantly diffuse and small-punctate spots of PHMB-FITC were observed, with a relatively small proportion of PHMB-FITC apparent within nuclei. To quantify PHMB entry into the cells, we analyzed treated cells using flow cytometry. Consistent with microscopy results, flow cytometry data indicate that PHMB-FITC entered approximately 70% of cells (**Figure 5B**).

### Colocalization of PHMB and Intracellular *S. aureus* in Mac-T Cells

In the previous experiment, we showed that PHMB killed intracellular *S. aureus* and entered Mac-T cells. To further understand possible interactions between PHMB and intracellular *S. aureus*, the host cells were infected with the bacteria and exposed to PHMB-FITC. Interactions between intracellular *S. aureus* and PHMB-FITC inside the host cells were viewed using confocal microscopy.

**FIGURE 6:**
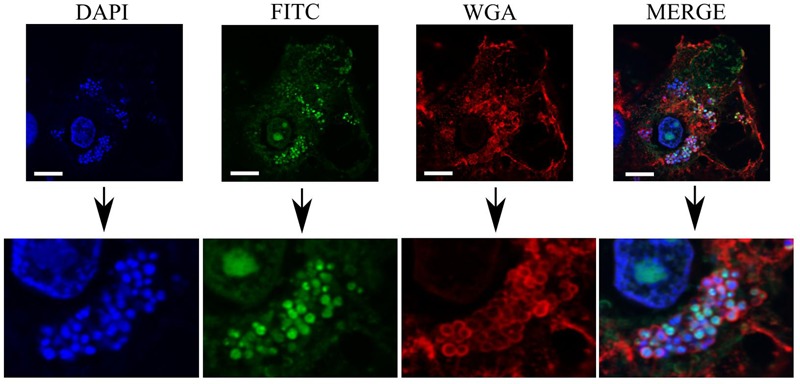
Colocalization of PHMB-FITC with intracellular *S. aureus* 15 AL inside Mac-T cells. Mac-T cells were infected with *S. aureus* strain 15 AL followed by treatment with PHMB-FITC (green). Prior to microscopy, cells were labeled with DAPI (blue) and WGA (red). Upper panels are images of infected cells visualized using filters for DAPI, FITC, and WGA, and merged images. Lower panels are enlarged images that clearly show colocalization between PHMB-FITC (green) and *S. aureus* 15AL (blue dots). White scale bar is 25 μM.

As can be seen in **Figure 6**, colocalization of blue-stained bacteria and green PHMB-FITC was apparent inside host cells, as indicated by red-stained membrane structures. Indeed, colocalization of PHMB-FITC and *S. aureus* in Mac-T cells was apparent for the majority of intracellular bacteria in all cells examined. Therefore, PHMB-FITC appears to directly access intracellular *S. aureus* inside the host cells, suggesting direct killing of intracellular bacteria in this cell culture model.

### Enrofloxacin and PHMB Toxicities toward Mac-T Cells

Resazurin assays were performed to determine the toxicity levels of enrofloxacin and PHMB against Mac-T cells. The half maximal inhibitory concentrations (IC_50_) for enrofloxacin and PHMB were 345 ± 91 and 21 ± 2 mg/L, respectively. Therefore, Mac-T cells tolerated both compounds at concentrations that were higher than the concentrations required to kill more than 90% of intracellular *S. aureus*; enrofloxacin (15 mg/L) and PHMB (15 mg/L). These results indicate that both compounds can be used to kill intracellular *S. aureus* with a high therapeutic index, at least in these *in vitro* model systems.

### Biofilms Formation and Visualization

**FIGURE 7:**
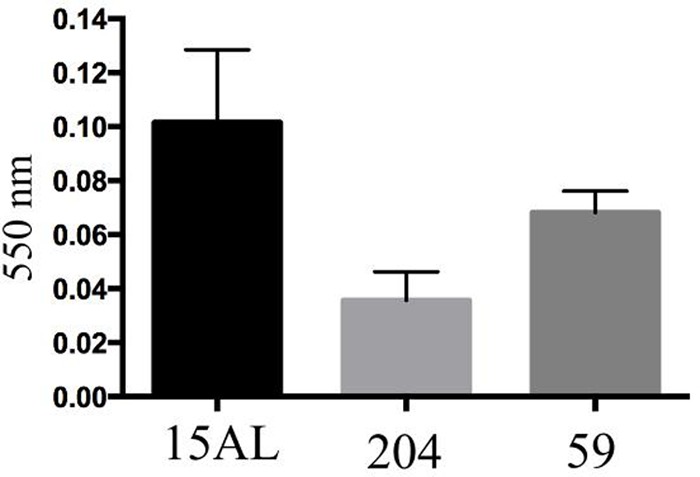
Biofilms’ mass of three *S. aureus* strains measured by crystal violet staining. Biofilms were grown for 48 h and stained with crystal violet. The biofilms’ mass was quantified by measuring the optical density of crystal violet at 550 nm using spectrophotometer.

**FIGURE 8:**
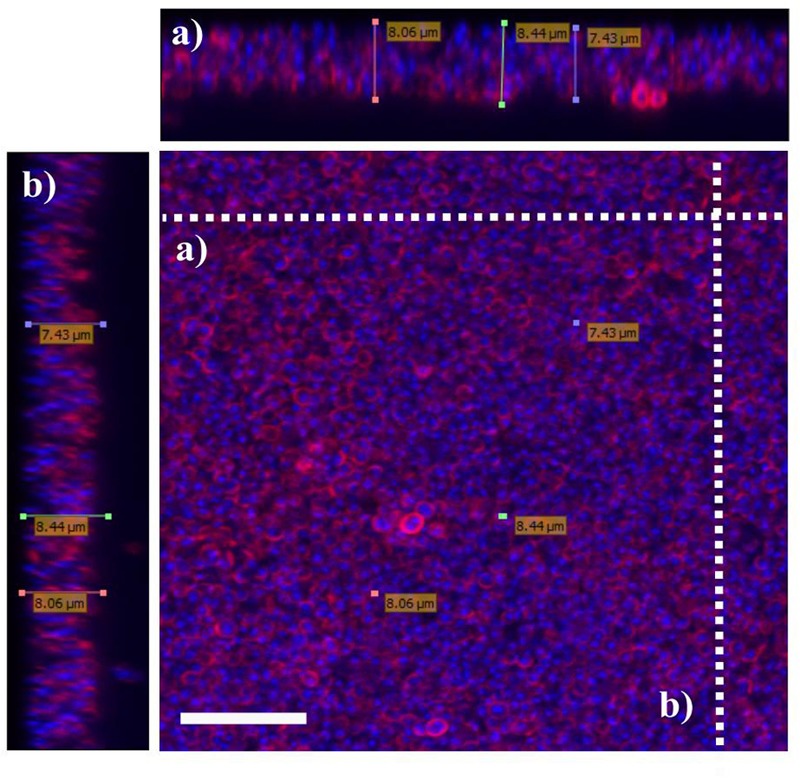
Visualization of *S. aureus* biofilms using confocal microscopy. Biofilms of *S. aureus* 15 AL were grown for 48 h. Prior to imaging, biofilms were stained with DAPI (blue) for *S. aureus* nuclei staining and WGA (red) for *N*-acetylglucose amine staining. Confocal microscopy *z*-stack projection moved through 111 slices across the cell. **(a)** Horizontal cross-section of biofilms **(b)** Vertical cross-section of biofilms. White scale bar is 7.5 μm.

To compare biofilm formation by *S. aureus* strains used in this study, the mass of biofilm produced by each strain were measured using crystal violet staining assay. We observed that incubation of the bacteria for 48 h produced the highest amount of biofilm as indicated by highest OD upon staining with crystal violet. Prolonging the incubation time to 72 h or more reduced the amount of the biofilm produced. Of all three strains tested; 15 AL, 204, and 59, strain 15AL produced the most biofilms (**Figure 7**). To further confirm the structure and thickness of biofilms, the biofilms of strain 15 AL were imaged using confocal microscopy. Using the three dimensional projection, we confirmed the structure of biofilms was composed of stacks of 5-7 bacteria grown on top of each other, with thickness was in between 7.9 ± 0.5 μm (**Figure 8**).

### Anti-biofilms Activities of Enrofloxacin and PHMB

**FIGURE 9:**
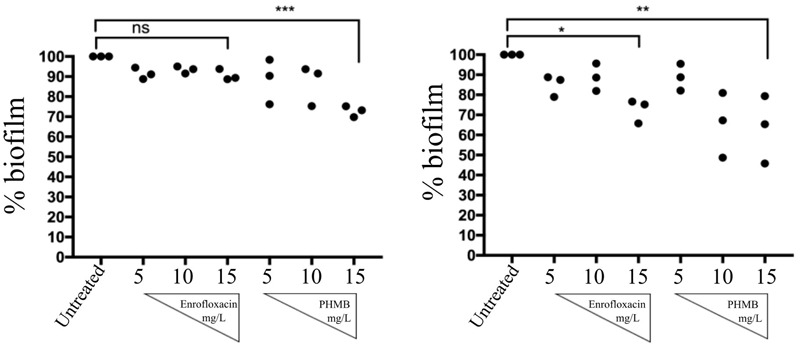
Anti-biofilms activities of enrofloxacin and PHMB. Biofilms of *S. aureus* strain 15 AL and 59 were either untreated or treated with increasing concentrations of enrofloxacin or PHMB. Untreated biofilms were used as a positive control, corresponding to 100% biofilms’ mass.

To investigate anti-biofilms activities of enrofloxacin and PHMB, biofilms were grown as previously described. Due to instability of the biofilms produced by strain 204, only biofilms produced by strain 15 AL and 59 were tested. After 48 h, biofilms of both strains were treated with enrofloxacin or PHMB at 5, 10, or 15 mg/L for 3 h and stained with crystal violet to expose mass of remaining biofilms. **Figure 9** shows percentages of biofilm mass after treatment with enrofloxacin or PHMB, relative to the untreated biofilms (100%). Enrofloxacin at 15 mg/L reduced 10 and 27% of biofilms *S. aureus* strain15 AL and 59, respectively. PHMB at 15 mg/L reduced 28 and 37% of biofilms 15 AL and 59, respectively. Therefore, while both antimicrobials displayed anti-biofilms activities, PHMB was more potent in disrupting the biofilms of both strains. In addition, both antimicrobials were effective in killing biofilm bacteria (Supplementary Figure S1).

## Discussion

Host cell invasion and the formation of biofilms are believed to be among the main factors leading to *S. aureus* mastitis treatment failures. Here we tested two antimicrobials, enrofloxacin and PHMB against intracellular and biofilms forms of *S. aureus.* We demonstrated that both enrofloxacin and PHMB killed intracellular *S. aureus* and displayed anti-biofilm activities. PHMB activities against intracellular *S. aureus* appears through direct interactions with the bacteria inside the host cells.

The low MIC values for both enrofloxacin and PHMB suggest that all internalized *S. aureus* are likely to be susceptible to killing by both compounds, provided the antimicrobial reaches bactericidal concentrations inside the host cells. Enrofloxacin is a fluoroquinolone that displays concentration dependent killing against extracellular bacteria and can enter mammalian cells (Schoevers et al., 1999). Thus, we anticipated that enrofloxacin would display the same killing activities against all intracellular *S. aureus.* However, variable activities and incomplete clearance of the bacteria indicates that the intracellular milieu reduces potency. Similarly, Schoevers et al. (1999) demonstrated that intracellular *S. aureus* in porcine alveolar macrophages are less susceptible to enrofloxacin, and Vallet et al. (2011) demonstrated incomplete clearance of intracellular *S. aureus* when treated with gemifloxacin. Furthermore, higher cellular drug accumulation did not always lead to higher intracellular activity, possibility due to drug binding non-specifically to intracellular components (Vallet et al., 2011).

PHMB in contrast, displayed bactericidal activities against all intracellular *S. aureus* tested. This finding agrees with our previous report of the bactericidal activities of PHMB against intracellular *S. aureus* in keratinocytes (Kamaruzzaman et al., 2016). In this and a related study on intracellular parasites, we found that that PHMB uptake into mammalian cells is through dynamin-dependent endocytic mechanism (Firdessa et al., 2015; Kamaruzzaman et al., 2016). The compound is first trapped within endosomes that could latter rupture due to two possible mechanisms; (1) The “flip-flop” mechanism, where interaction of the positive charge of the cationic biguanide can cause anionic phospholipid from cytosol leaflet to flip into intraluminal side of the endosome, thus causing destabilization of the structure and released of the cargo into the cytosol or, (2) The “proton-sponge effect” where protonation of PHMB in acidic endolysosome can induces extensive inflow of ions and water into the compartments, subsequently causing rupture of the membrane and released of the compound into the cytosol (Varkouhi et al., 2011). Once released into the cytosol, the compound could then enter the nuclei, although most of the polymer appears to be excluded. The predominantly diffuse and some punctate localization of PHMB-FITC inside the host cells suggests that this compound may have accessed to the cytosol and endosomal compartments, where *S. aureus* would normally localize, similar to our previous observations in keratinocytes. However, higher concentrations of PHMB (15 mg/L) were required to kill intracellular *S. aureus* in bovine mammary epithelial cells compared to intracellular *S. aureus* in keratinocytes (4 mg/L), suggesting that changes in bacterial physiology in these host cells or host cells factors may inhibit PHMB activities (Kamaruzzaman et al., 2016). This is supported by our observations of PHMB-FITC uptake in only 70% of Mac-T cells, compared to 99% in keratinocytes (Kamaruzzaman et al., 2016). Nonetheless, colocalization of PHMB with the majority of *S. aureus* inside bovine mammary epithelial cells suggests that PHMB enters Mac-T cells, and then directly binds and kills intracellular bacteria.

PHMB’s antibacterial activities appear to be due, in part, to the biguanide groups interacting with cytoplasmic membranes, lipopolysaccharidse and peptidoglycan of the bacterial cell wall. This binding could displace the divalent cation Ca^2+^, causing membrane destabilization and cellular leakages (Gilbert and Moore, 2005). Also, the hexamethylene segment can interact with phospholipids on the membrane, causing a phase separation that disturbs random distribution of lipids, further destabilizing the membrane structure (Broxton et al., 1984). Furthermore, recent findings in our laboratory demonstrated that PHMB can enter bacteria cells and condense bacterial chromosomes (Chindera et al., 2016). PHMB has a strong affinity toward DNA which is believe due to the strong electrostatic interaction between the negatively charged phosphate backbone of DNA and the cationic charged of polymer PHMB (Firdessa et al., 2015). Therefore, PHMB’s antimicrobial activities involve several mechanisms.

In this study, enrofloxacin was only effective in disrupting the biofilms from one of the two strains tested, suggesting strain dependent anti-biofilms activities. Variability between biofilms of different *S. aureus* strains treated by a delafloxacin (a fluoroquinolone) was also reported by Siala et al. (2014). The study suggests that different chemical compositions and biophysical properties of the biofilms associated with different strains affects the penetration of the enrofloxacin inside the biofilms (Siala et al., 2014). In contrast, PHMB was more effective in disrupting biofilms structure, despite being a larger molecule. We cannot provide a detailed explaination of PHMB effects on biofilms; however, PHMB is a positively charged polymer that can interact with DNA through electrostatic interactions (Firdessa et al., 2015). Biofilms are composed of self-produced extracellular polymeric substances (EPS) that consist of polysaccharide, extracellular DNA, protein and lipids. The role of extracellular DNA is to promote adhesion of the bacteria to the surfaces and to provide structural integrity to the EPS structure (Okshevsky and Meyer, 2013). Thus, the anti-biofilms effects we observed could be due to interactions between PHMB with the EPS structure.

## Conclusion

There are three main findings from this study. First, both enrofloxacin and PHMB are effective against intracellular *S. aureus*. Second, PHMB directly binds to intracellular *S. aureus* in the Mac-T cells. Third, both compounds also have anti-biofilm activities. These findings demonstrate that PHMB is an effective antimicrobial polymer against intracellular and biofilm forms of *S. aureus*, and this polymer appears to have potential as a future therapy for the treatment of mastitis, a disease that involves difficult to treat *S. aureus* infections.

## Author Contributions

NK and LG designed the research. NK, SC, KE-B, WN-B performed the experiment, NK, SC, and LG wrote the manuscript, MB provided the strains and technical advice on development of biofilms.

## Conflict of Interest Statement

The patent for PHMB application has been filed. The assignment is owned by Royal Veterinary College. The authors declare that the research was conducted in the absence of any commercial or financial relationships that could be construed as a potential conflict of interest.
