# Supplementary material for: Bactericidal and Anti-biofilm Effects of Polyhexamethylene Biguanide in Models of Intracellular and Biofilm of Staphylococcus aureus Isolated from Bovine Mastitis
Source: Front Microbiol. 2017 Aug 11;8:1518. doi: 10.3389/fmicb.2017.01518 (PMC5554503; doi:10.3389/fmicb.2017.01518)
Supplement: Supplementary file 1 [file Data_Sheet_1.DOCX]

Supplementary Material

Bactericidal and anti-biofilm effects of polyhexamethylene biguanide in models of intracellular and biofilm of *Staphylococcus aureus* isolated from bovine mastitis

**Nor Fadhilah Kamaruzzaman^1,‡2*^ Stacy Qian Yu Chong^1^, Kamina Monika Edmondson-Brown^1^, Winnie Ntow-Boahene^1^, Marjorie Bardiau^3^ & Liam Good^1^**

*** Correspondence:** Corresponding Author: norfadhilah@umk.edu.my


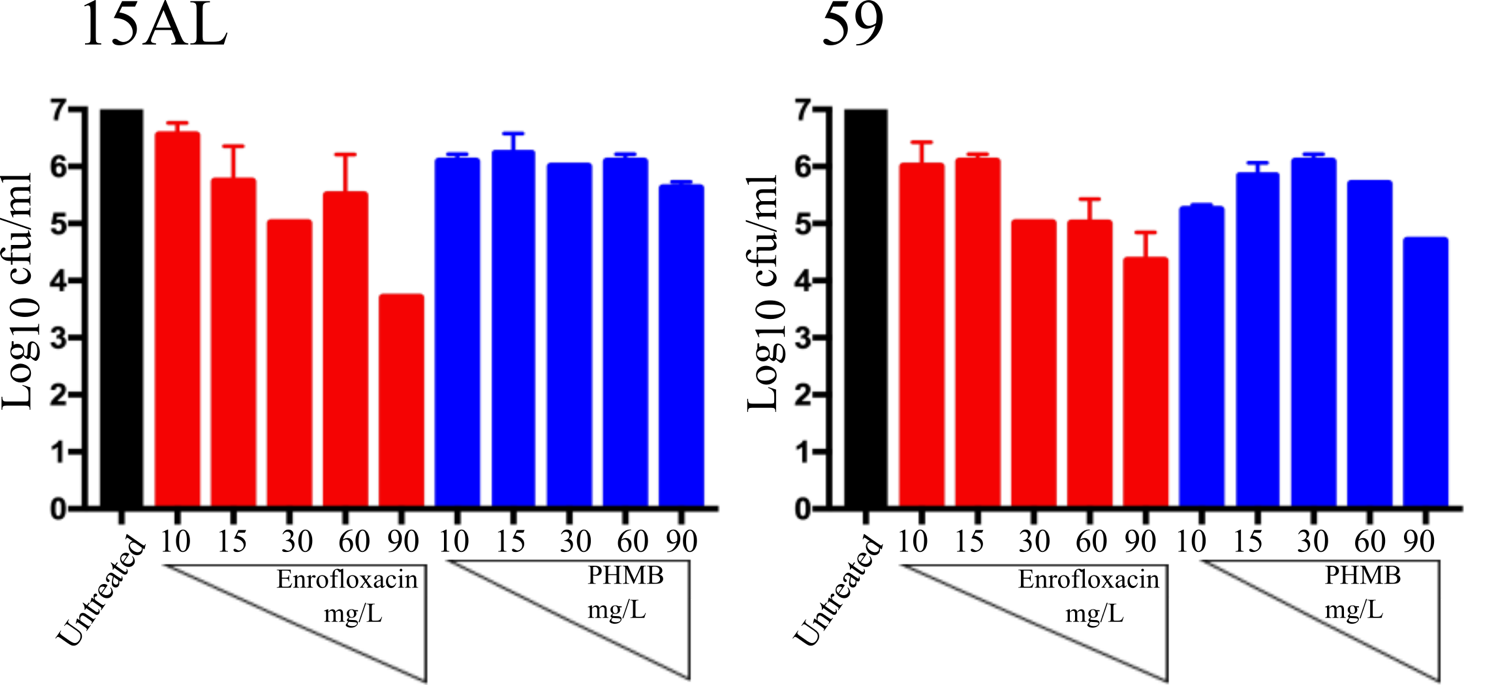


Supplementary Figure 1 Bactericidal activities of enrofloxacin and PHMB against biofilms of *S. aureus* 15 AL and 59

Biofilms of *S. aureus* strain 15 AL and 59 were either untreated or treated with increasing concentrations of enrofloxacin or PHMB and plated on nutrient agar for colony counting. The graph was generated from duplicate cfu values.
